# Inflammasome–Interferon Convergence in Elite HIV Controllers and Autoimmune Diseases: A Critical Integrative Reflection

**DOI:** 10.3390/diseases14080304

**Published:** 2026-08-21

**Authors:** Ariel Torres, Paloma González, Martha Fors, Gisselle Trujillo

**Affiliations:** 1 Facultad de Ciencias de la Salud y Bienestar Humano, Universidad Tecnológica Indoamérica, Quito 170103, Ecuador; arieltorres@uti.edu.ec (A.T.); gisselletrujillo@uti.edu.ec (G.T.); 2 Biblioteca, Universidad de Las Américas, Quito 170125, Ecuador; 3 One Health Research Group, Facultad de Ciencias de la Salud, Universidad de Las Américas, Quito 170125, Ecuador; martha.fors@udla.edu.ec

**Keywords:** elite HIV controllers, autoimmune diseases, innate immunity, inflammasome, type I interferon, chronic inflammation

## Abstract

Background: Elite HIV controllers exhibit persistent immune activation despite sustained viral suppression, a phenomenon that challenges the traditional view of immunological equilibrium. In parallel, autoimmune diseases are characterised by chronic inflammation driven by dysregulated immune responses. Objective: This study aimed to analyse the potential pathophysiological convergence between both conditions and to identify a shared inflammatory axis with possible translational implications. Methods: A critical narrative and integrative reflection was conducted using a purposive, concept-driven selection of evidence from PubMed/MEDLINE, Scopus, and Web of Science. A total of 43 sources were included: 28 studies informing mechanistic findings (14 on elite HIV controllers and 14 on autoimmune diseases), 12 addressing modulatory strategies (pharmacological and lifestyle-based), and 3 providing contextual frameworks. Results: Both conditions demonstrate sustained activation of innate immunity, characterised by elevated pro-inflammatory cytokines (IL-1β, IL-6, TNF, type I interferons), increased inflammatory biomarkers (e.g., sCD14, IP-10), and activation of pathways such as NLRP3 inflammasome and NF-κB signalling. Collectively, these findings suggest convergence towards a potential shared inflammatory axis mediated by the inflammasome–interferon pathway, which may contribute to persistent systemic inflammation and tissue damage. Evidence from pharmacological and non-pharmacological interventions suggests that components of this axis may be susceptible to modulation. Conclusions: The observed convergence supports a conceptual model of shared innate inflammatory activation across both conditions. Rather than implying a unified therapeutic approach, the findings suggest a framework of convergent modulation targeting the inflammasome–interferon axis. This hypothesis warrants further investigation to determine its clinical and translational relevance.

## 1. Introduction

Chronic inflammation has increasingly been recognised as a cross-cutting biological process underlying a wide range of diseases, extending beyond traditional diagnostic boundaries. In the context of HIV infection, global health organisations have emphasised that, despite the effectiveness of antiretroviral therapy in achieving viral suppression, persistent immune activation remains a major driver of long-term morbidity and mortality, particularly through its association with cardiovascular and metabolic complications [1]. At the same time, autoimmune diseases are characterised by sustained immune dysregulation and chronic inflammation, representing a growing global health burden that requires deeper mechanistic understanding [2].

Elite HIV controllers represent a particularly intriguing population within this landscape. These individuals are able to maintain viral suppression in the absence of antiretroviral therapy, suggesting a highly effective immune response. However, emerging evidence indicates that this apparent control does not equate to immunological quiescence. On the contrary, persistent immune activation has been documented even in these patients, raising important questions about the biological cost of this state and its long-term consequences.

In parallel, advances in the understanding of autoimmune diseases have highlighted the central role of innate immune activation, particularly through pathways involving inflammasomes and type I interferon signalling. These mechanisms are now recognised as key drivers of chronic inflammation and tissue damage across multiple conditions. International frameworks have increasingly stressed the importance of addressing these shared inflammatory processes as part of a broader strategy to improve outcomes in chronic immune-mediated diseases [3].

Despite these advances, research on elite HIV controllers and autoimmune diseases has largely evolved in parallel, with limited integration between the two fields. This has resulted in a critical gap in knowledge: the possibility of a shared pathophysiological axis underlying persistent inflammation across these conditions remains insufficiently explored. In particular, it is unclear whether the mechanisms sustaining immune activation in elite HIV controllers overlap with those driving autoimmune processes and whether such convergence may have clinical or translational implications.

In this context, the present study aims to analyse the potential pathophysiological convergence between chronic inflammation in elite HIV controllers and autoimmune diseases, with a specific focus on identifying a shared inflammatory axis. By integrating mechanistic evidence and exploring potential modulatory strategies, this work seeks to contribute to a more comprehensive understanding of inflammation as a common biological process and to generate hypotheses with potential clinical and translational relevance.

## 2. Materials and Methods

This study was conducted using a critical narrative approach with an integrative orientation and a hypothesis-generating purpose, aimed at analysing the potential pathophysiological convergence between the chronic inflammation observed in elite HIV controllers and the persistent inflammatory mechanisms described in autoimmune diseases. This design was selected because the research question was not intended to estimate quantitative associations or evaluate specific interventions, but rather to interpret complex biological processes, contrast pathophysiological models, and generate clinically and translationally relevant hypotheses.

This work was therefore not conceived as an exhaustive review of all available evidence but as a concept-driven critical synthesis aimed at identifying biologically plausible points of convergence between two distinct immune-mediated scenarios. The selection and organisation of evidence were guided by conceptual relevance, mechanistic depth, and translational value, rather than by comprehensive coverage or quantitative aggregation.

The search for evidence was carried out in high-impact biomedical databases, including PubMed/MEDLINE, Scopus, and Web of Science, and was complemented by manual screening of reference lists. Search terms in both English and Spanish were combined using Boolean operators and included “HIV elite controllers”, “chronic inflammation”, “immune activation”, “autoimmune diseases”, “inflammasome”, “monocyte activation”, and “type I interferon”. The aim was to capture relevant literature addressing shared immunological mechanisms. Priority was given to publications from 2015 to 2026, including systematic reviews, high-level narrative reviews, translational studies, clinical cohorts, and original research with a focus on pathophysiology. Earlier seminal studies were included where necessary to contextualise key concepts.

Study selection followed a purposive and concept-driven approach. A total of 28 studies (14 focused on elite HIV controllers and 14 on autoimmune diseases) were included to characterise the shared inflammatory framework identified in the Results section. In addition, 12 studies were incorporated to explore potential modulatory strategies, including 7 pharmacological and 5 lifestyle-based interventions. Finally, 3 complementary sources, including guidelines and conceptual frameworks, were used to contextualise the Introduction. Selection prioritised high-level evidence, including systematic reviews, meta-analyses, and mechanistic or translational studies published in high-impact journals. In cases of overlapping evidence, priority was given to studies with greater mechanistic depth, translational relevance, methodological robustness, and consistency with recurrent inflammatory pathways identified across the literature.

**Figure 1:**
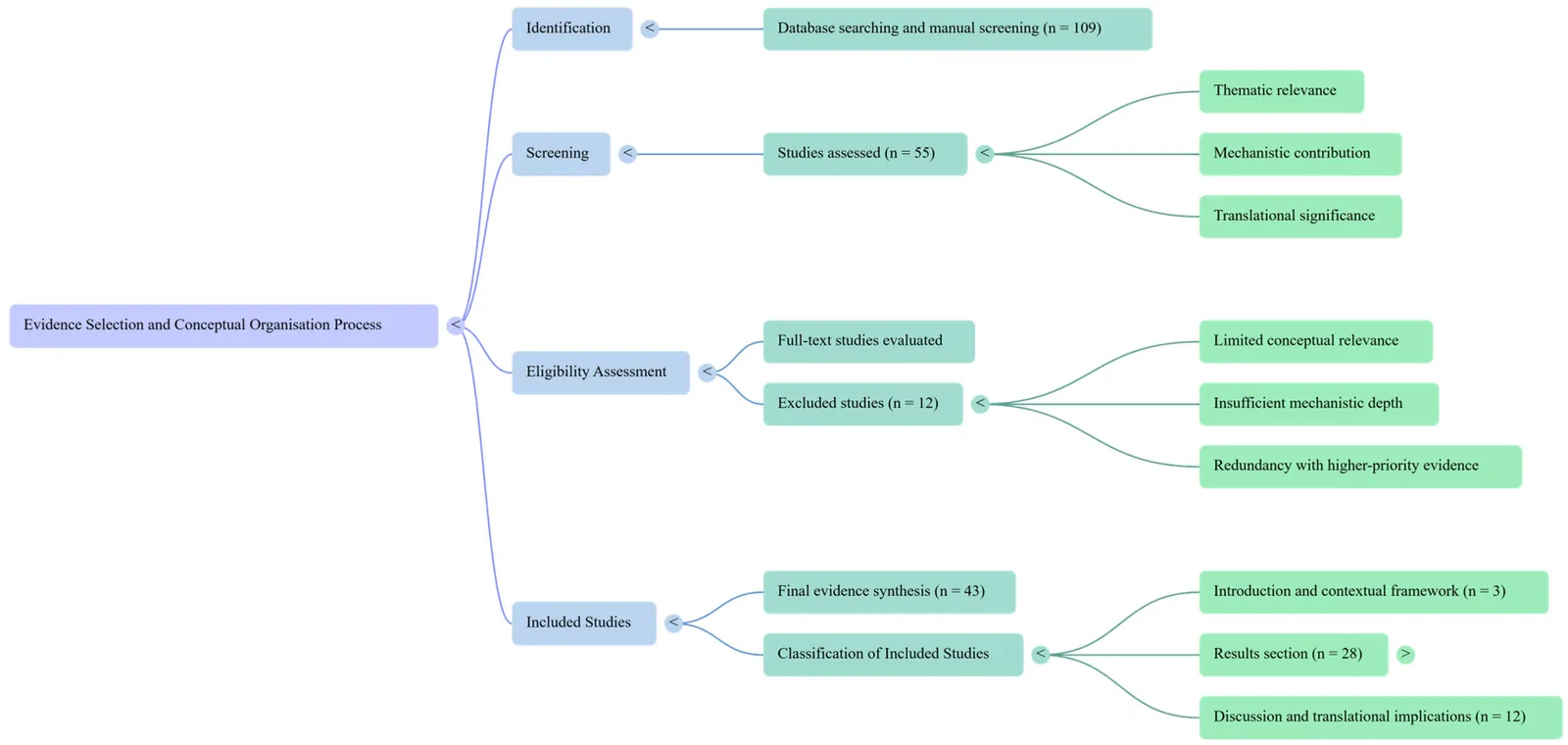
Evidence selection and conceptual organisation process. A total of 109 records were initially identified through database searching and manual screening. Following conceptual assessment and eligibility evaluation, 43 studies were included and organised according to their contribution to the Introduction, Results, and Discussion sections.

Studies focused exclusively on viral suppression under antiretroviral therapy without relevance to elite controllers, as well as those lacking mechanistic depth, were excluded. A simplified study selection flow diagram was constructed to enhance transparency in the identification, screening, and conceptual organisation of the evidence. This diagram does not follow a formal PRISMA framework, as the present study is a critical narrative and integrative reflection rather than a systematic review (Figure 1).

To enhance methodological transparency, study selection and classification were conducted through an iterative consensus process among the authors. Initially, potentially relevant studies were identified based on their thematic alignment with the research objective. Subsequently, full-text screening was performed to assess conceptual relevance, mechanistic contribution, and translational value. Discrepancies in study inclusion or classification were discussed among the authors until agreement was reached. Although formal inter-rater reliability was not calculated due to the narrative nature of the study, efforts were made to ensure consistency and minimise subjective bias throughout the selection process.

The analysis was conducted through a comparative thematic synthesis aimed at identifying convergent patterns between both conditions. First, the predominant inflammatory mechanisms in elite HIV controllers were characterised, including persistent innate immune activation, monocyte–macrophage dysfunction, interferon signalling, and potential microbial translocation. In parallel, the principal pathways involved in autoimmune diseases were analysed, including loss of immune tolerance, Toll-like receptor activation, inflammasome involvement, and sustained production of pro-inflammatory cytokines. Based on this comparative framework, an integrative conceptual axis was constructed to identify a potential shared pathophysiological node underlying chronic inflammation in both scenarios.

Subsequently, a critical interpretative analysis was undertaken with the aim of generating hypotheses, considering the potential existence of shared modulators, amplifiers, or regulatory mechanisms. Any therapeutic implications derived from this analysis were formulated strictly as theoretical hypotheses for future research and were not intended for direct clinical application, in line with the exploratory nature of the study.

Methodological rigour was ensured through the prioritisation of high-level evidence, the explicit differentiation between empirical findings and interpretative propositions, and the acknowledgment of limitations related to the heterogeneity of elite HIV controllers and the diversity of autoimmune diseases. As this study was based on documentary analysis, ethical approval was not required. All procedures adhered to the principles of scientific integrity and appropriate citation of sources.

Artificial intelligence-assisted tools were used exclusively for language refinement and the visual development of conceptual figures. These tools did not contribute to study selection, data extraction, analysis, or interpretation. All methodological decisions and conceptual development were conducted solely by the authors in accordance with established principles of scientific integrity.

## 3. Results

### 3.1. Elite HIV Controllers

(1) Persistent immune activation and inflammatory biomarkers

Elite controllers exhibit persistent innate immune activation despite suppression of plasma viraemia, evidenced by elevated levels of D-dimer, soluble tissue factor, IL-1β, C-reactive protein, and IL-6, along with induction of interferon-stimulated genes [4]. Elevated levels of soluble inflammatory markers such as sCD14, IFN-γ, IP-10, and IL-10 have been documented, with concentrations approximately doubled compared to individuals with HIV receiving antiretroviral therapy and uninfected controls [5]. Additionally, biomarkers such as sTNF-R1, IFN-α, and IP-10 have been identified as being associated with loss of viral control in elite controllers [6]. Recent reviews describe the persistence of chronic systemic immune activation even in the absence of detectable viraemia [7,8].

(2) Specific cellular and immunological alterations

An increase in γδ T cells (Vδ1+) with a pro-inflammatory profile and production of IFN-γ, TNF-α, and MIP-1β has been observed in elite controllers, with a correlation between their frequency and viral RNA levels in intestinal tissue [9]. Persistent activation of innate immunity has also been described, including the involvement of CD8 cells and associated genetic variability (CCR5, HLA) [10]. In parallel, disruption of monocyte homeostasis has been reported in long-term elite controllers [11]. Furthermore, NK cells exhibit an activated and mature phenotype, with low exhaustion but altered functional activity [12].

(3) Residual inflammation and associated comorbidities

A higher prevalence of coronary atherosclerosis has been documented in elite controllers compared to HIV-negative controls, along with increased levels of sCD163 as a marker of monocyte activation [13]. Subsequent studies confirm the presence of residual inflammation associated with cardiovascular risk even in the absence of detectable viraemia [14]. Chronic inflammation in HIV has been linked to a 1.5–2-fold increase in cardiovascular risk [15]. Additionally, cardiometabolic alterations related to persistent systemic inflammation have been described [8].

(4) Immunological heterogeneity and mechanisms of viral control

Differences have been identified between persistent and transient elite controllers, associated with the size of the viral reservoir and the quality of the immune response [16]. So-called “exceptional elite controllers” exhibit prolonged viral suppression associated with specific host immunological and genetic interactions [17]. Overall, studies indicate that viral control in these individuals is maintained through a coordinated response of both innate and adaptive immunity [7,18].

### 3.2. Interferon Axes and Sustained Immune Activation

(1) NLRP3 inflammasome and central inflammatory signalling

Inflammasomes, particularly NLRP3, are described as central axes of inflammation in autoimmune diseases, mediating the activation of IL-1β and IL-18 and contributing to chronic tissue damage [19,20,21]. The NLRP3 inflammasome acts as a key regulator of innate immunity and is directly involved in conditions such as lupus, rheumatoid arthritis, and inflammatory bowel disease [22]. Activation of this pathway in dendritic cells and macrophages further amplifies inflammatory responses through enhanced cytokine production and sustained tissue injury [23]. In addition, inflammasome signalling platforms mediate caspase-1 activation and the maturation of pro-inflammatory cytokines, reinforcing persistent inflammatory activity [24].

(2) Interferon axes and sustained immune activation

The type I interferon axis is identified as a central mechanism in autoimmunity, particularly in lupus, where it regulates sustained immune activation and clinical stratification [25,26]. Type I interferon also acts as both an initiator and amplifier of the autoimmune response, associated with loss of immune tolerance and persistent activation of B and T lymphocytes [27]. In addition, the STING–inflammasome axis has been described as an integrator of immune signals, linking nucleic acid sensing to inflammation and chronic tissue damage [28].

(3) Dysregulation of innate immunity and loss of tolerance

Autoinflammatory diseases arise from dysregulation of innate immunity, involving inflammasomes, NF-κB, TNF, and IL-1, leading to persistent antigen-independent inflammation [29]. Likewise, loss of immune tolerance in T and B lymphocytes is recognised as a central feature of autoimmunity, accompanied by aberrant cytokine production, sustained pathogenic T-cell responses, and progressive amplification of inflammatory signalling cascades [30,31]. Autoimmunity has been shown to result from the interaction between genetic and environmental factors and immune dysfunction, with involvement of pro-inflammatory cytokines such as TNF, IL-1, IL-6, and IFN-γ in the perpetuation of tissue damage [30,31,32].

(4) Chronic inflammation, inflammaging, and tissue damage

Autoimmune diseases are characterised by persistent inflammation and loss of immune tolerance, associated with immunological ageing or “inflammaging”. Chronic activation of the inflammasome, particularly NLRP3, induces a sustained inflammatory state even in younger patients [33]. This persistent inflammatory state is associated with continuous secretion of pro-inflammatory cytokines and progression of tissue damage across multiple organs [19,24].

### 3.3. Shared Inflammatory Framework

Studies involving elite HIV controllers and autoimmune diseases consistently describe sustained innate immune activation associated with increased levels of pro-inflammatory cytokines, including IL-1β, IL-6, TNF, and type I interferons [4,5,19,25]. In parallel, elevated inflammatory biomarkers such as IP-10, sCD14, and sTNF-R1 have been identified across both conditions [5,6,24].

Evidence also demonstrates activation of innate immune cell populations, including monocytes, macrophages, and NK cells, together with persistent production of inflammatory mediators [9,10,12,23]. Similarly, activation of inflammasome pathways—particularly NLRP3—has been associated with IL-1β and IL-18 release in both disease contexts [19,20,21,24].

Sustained activation of the type I interferon axis has likewise been linked to persistent immune dysregulation and chronic inflammatory responses [6,25,27]. Across both conditions, studies also report chronic systemic inflammation associated with continuous production of pro-inflammatory mediators and progressive tissue injury [13,15,32,33]. In addition, dysregulated inflammatory signalling involving NF-κB, TNF, and cytokine-related pathways has been described in both scenarios [29,30,31].

**Figure 2:**
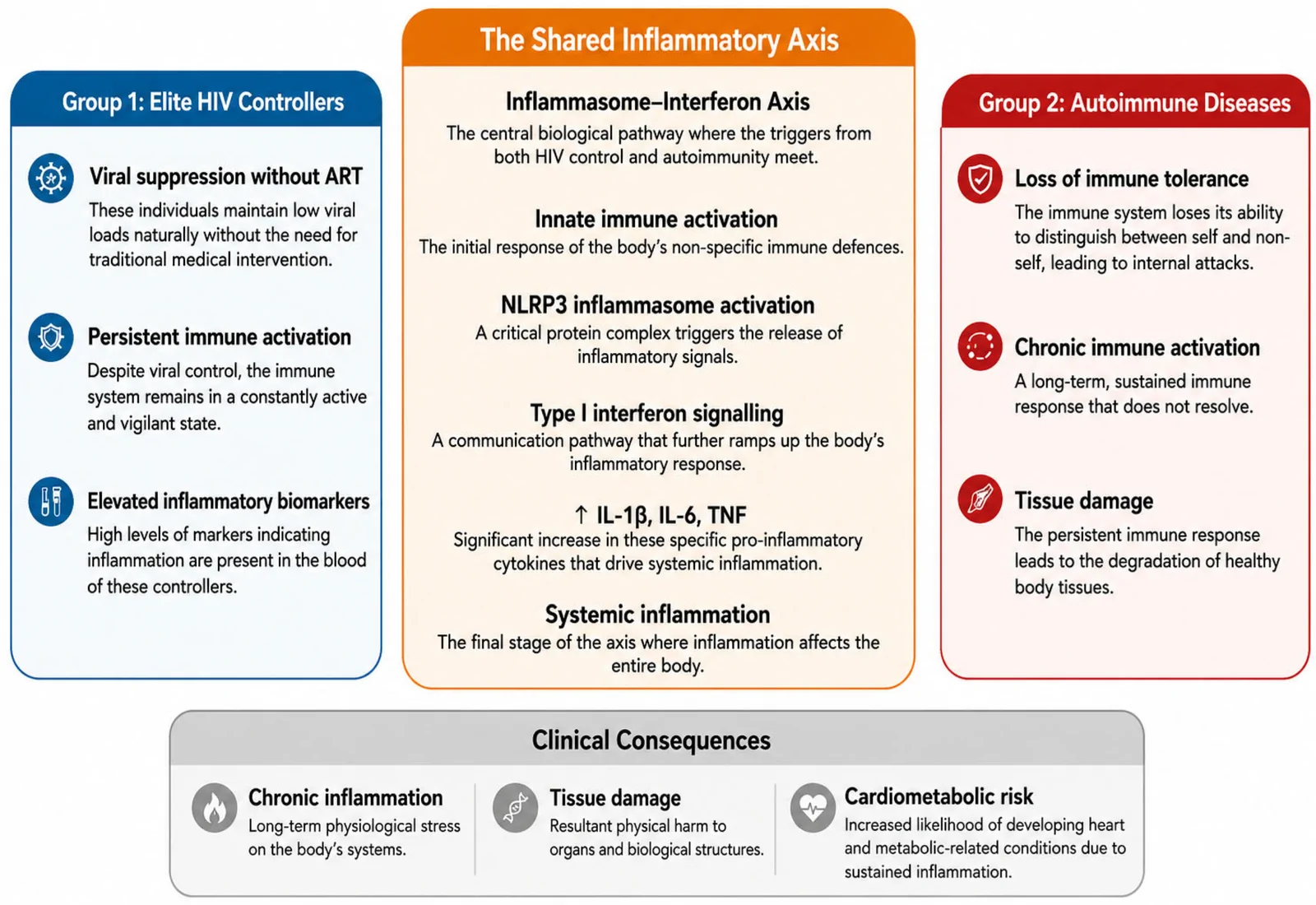
Inflammasome–interferon axis as a shared inflammatory pathway in elite HIV controllers and autoimmune diseases.

Overall, the available evidence supports the existence of partially overlapping inflammatory patterns characterised by inflammasome involvement, sustained type I interferon signalling, and persistent innate immune activation across both conditions (Figure 2). Collectively, these findings reinforce the biological plausibility of a shared inflammatory framework linking elite HIV controllers and autoimmune diseases.

This figure illustrates the proposed convergence between elite HIV controllers and autoimmune diseases through shared innate immune activation pathways, particularly NLRP3 inflammasome activation and type I interferon signalling. The model highlights the involvement of pro-inflammatory cytokines, innate immune cell activation, and downstream inflammatory consequences, including tissue damage and cardiometabolic complications.

To facilitate comparative interpretation of the evidence identified across both conditions, Table 1 summarises the principal shared inflammatory and immunological features described in elite HIV controllers and autoimmune diseases. The table integrates the main convergent mechanisms reported in the included studies, particularly those related to persistent innate immune activation, inflammasome signalling, type I interferon pathways, cytokine dysregulation, and chronic systemic inflammation. Additionally, it highlights the potential translational relevance of these overlapping inflammatory patterns within the context of emerging immunomodulatory approaches.

## 4. Discussion

For years, elite HIV controllers have been regarded as a model of near-ideal immunological equilibrium. However, accumulating evidence necessitates a more nuanced interpretation. Rather than representing a state of “silent” immunity, these individuals exhibit persistent immune activation, characterised by elevated levels of pro-inflammatory cytokines and inflammatory biomarkers, even in the absence of detectable viraemia [4,5,7,18]. What initially appears paradoxical becomes more coherent when analysed alongside mechanisms described in autoimmune diseases. Importantly, elite HIV control is biologically heterogeneous and may result from multiple, non-mutually exclusive mechanisms involving innate and adaptive immune responses, viral factors, and host-related determinants. An additional possibility is that persistent innate immune activation in both settings may be influenced, at least partially, by microbial translocation and sustained exposure to endogenous or exogenous inflammatory stimuli, although the relative contribution of these mechanisms remains uncertain.

From a mechanistic perspective, this inflammatory state has been associated with monocyte activation, interferon-stimulated gene expression, and inflammasome-related cytokine production. Nevertheless, an important unresolved question remains whether this sustained activation represents a biological cost of viral control, a compensatory antiviral mechanism, or a combination of both processes. This uncertainty highlights the complexity of interpreting immune activation in elite HIV controllers and cautions against simplistic pathogenic interpretations. A plausible interpretation is that these mechanisms are not mutually exclusive. An initially effective interferon–inflammasome response may contribute to viral containment, whereas persistent activation of the same pathways over time may subsequently promote chronic inflammation and inflammatory comorbidities. Although this conceptual sequence remains hypothetical, it may help explain the coexistence of effective viral control and persistent immune activation in elite HIV controllers.

In autoimmune diseases, by contrast, inflammation constitutes a central component of the pathological process itself. Activation of the inflammasome—particularly NLRP3—together with type I interferon signalling, promotes a self-sustaining inflammatory environment associated with progressive tissue damage [19,21,25]. Beyond its intensity, the defining feature of this response is its persistence and capacity for self-amplification.

When both scenarios are examined in an integrated manner, a recurrent point of convergence emerges across the available evidence. Elite HIV controllers and autoimmune diseases both exhibit activation of innate immune cell populations, including monocytes, macrophages, and NK cells, together with sustained production of pro-inflammatory cytokines and type I interferon mediators [9,12,27]. This inflammatory profile is further reinforced by intracellular signalling pathways involving NF-κB, TNF, and related cytokine networks, which contribute to the perpetuation of inflammatory responses [29,30,31].

Rather than implying biological equivalence, these findings suggest partially overlapping innate immune programmes involving inflammasome activation, type I interferon signalling, and chronic cytokine production. Rather than functioning as isolated pathways, these mechanisms likely interact within interconnected inflammatory networks that sustain chronic inflammatory activity across distinct disease contexts. Importantly, overlapping inflammatory signatures should not be interpreted as evidence of identical pathogenesis, as similar innate immune profiles may emerge from biologically distinct initiating mechanisms. Nevertheless, the recurrence of these pathways supports the biological plausibility of a partially shared inflammatory framework linking elite HIV controllers and autoimmune diseases. Importantly, this proposed convergence appears to be most relevant to systemic autoimmune diseases characterised by prominent type I interferon signatures, particularly systemic lupus erythematosus and Sjögren’s syndrome, rather than uniformly across all autoimmune conditions.

Genetic factors may also contribute to this proposed convergence. Several HLA alleles have been associated with both elite HIV control and susceptibility to specific autoimmune diseases. For example, HLA-B27 has been linked to effective control of HIV replication and is also a recognised genetic risk factor for ankylosing spondylitis, whereas HLA-DR4 has been associated with particular elite-controller phenotypes and rheumatoid arthritis. These observations suggest that host immunogenetic background may influence the balance between effective immune surveillance and persistent inflammatory activation.

At this stage, the discussion moves beyond descriptive comparison towards potential translational implications. If a partially shared inflammasome–interferon axis exists across both conditions, it becomes reasonable to consider whether this pathway may also be susceptible to therapeutic modulation. Available evidence suggests that this may be possible, at least partially. In autoimmune diseases, therapies targeting type I interferon signalling, such as anifrolumab, have demonstrated clinical efficacy together with reductions in inflammatory signatures [26,34,35]. Likewise, strategies directed at plasmacytoid dendritic cells, including litifilimab, have shown the capacity to modulate interferon-related pathways and improve disease activity [36]. Modulation of intracellular signalling pathways such as JAK/BTK has also demonstrated effects on systemic inflammatory responses [37].

Nevertheless, these findings should be interpreted within their specific disease contexts and should not be directly extrapolated to elite HIV controllers without dedicated mechanistic and clinical validation. In HIV research, the available evidence remains more limited but not entirely absent. Aspirin has been associated with reductions in immune activation markers such as sCD14 and tissue factor in elite controllers, while atorvastatin has demonstrated effects on inflammatory cytokines and cellular activation [38]. Although these findings remain exploratory and do not support routine clinical use of anti-inflammatory interventions in elite HIV controllers, any attempt to modulate these pathways should be approached cautiously, as excessive suppression of antiviral immune responses could potentially compromise mechanisms involved in natural viral control.

An additional dimension emerges from non-pharmacological approaches. Interventions such as physical exercise, dietary patterns, and nutritional strategies have consistently demonstrated anti-inflammatory effects across different chronic conditions. Regular exercise has been associated with reductions in IL-6, TNF-α, C-reactive protein, IL-1β, and IL-18, mediators closely linked to inflammasome activity [39,40]. Similarly, the Mediterranean diet and time-restricted eating have shown beneficial effects on systemic inflammatory markers, including TNF-α [41,42]. In individuals living with HIV, resistance training has also been associated with improvements in immuno-inflammatory profiles [43]. Although the direct impact of these interventions on the proposed shared inflammatory framework remains uncertain, the consistency of their anti-inflammatory effects further supports the biological relevance of chronic innate immune activation across both conditions.

**Figure 3:**
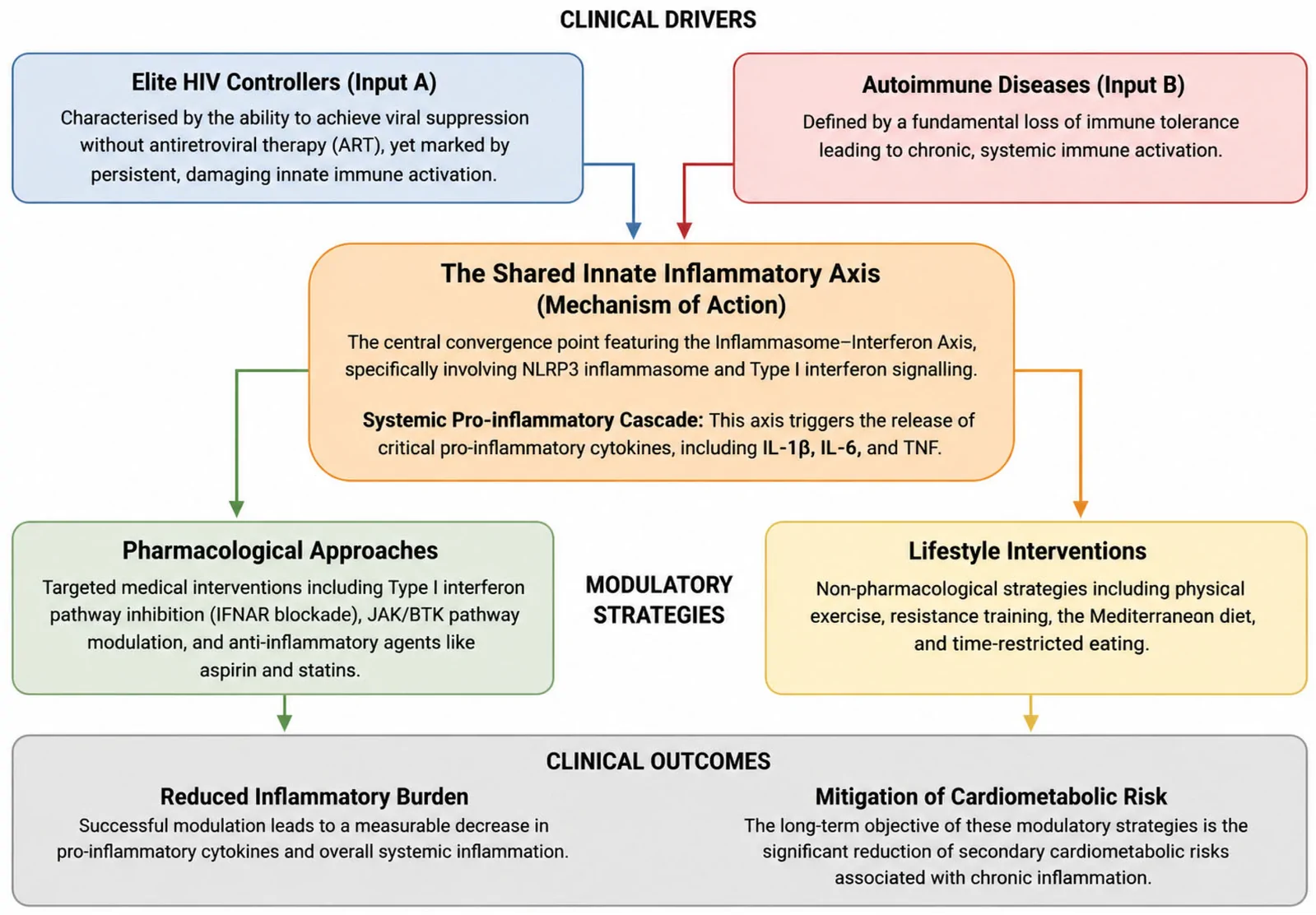
Conceptual framework of potential modulatory strategies targeting the shared innate inflammatory axis.

Collectively, these findings support a more nuanced interpretation of the proposed inflammatory convergence. The issue is not the direct transfer of therapeutic strategies from one condition to another, but rather the recognition of partially shared inflammatory pathways that may require context-specific modulation (Figure 3). In autoimmune diseases, suppression of immune activation may be therapeutically beneficial; in elite HIV controllers, however, any intervention must preserve the delicate immunological balance that contributes to viral containment. In this context, immune activation in HIV cannot be interpreted as exclusively pathological, as it may also represent part of a functional antiviral response.

This duality highlights the context-dependent biological significance of persistent innate immune activation. In autoimmune diseases, chronic inflammation predominantly contributes to tissue injury and loss of immune tolerance, whereas in elite HIV controllers, similar inflammatory pathways may simultaneously participate in antiviral defence while also contributing to systemic inflammatory burden. Consequently, partially overlapping inflammatory signatures do not necessarily imply equivalent pathogenic roles across both conditions.

This figure presents a conceptual framework of potential modulatory strategies targeting the proposed shared inflammatory axis. It illustrates a gradient of intervention, ranging from non-pharmacological approaches (such as physical activity and dietary patterns) to pharmacological strategies with indirect anti-inflammatory effects and finally to targeted modulation of specific pathways, including interferon signalling and inflammasome activation. The framework emphasises the need for differential application across disease contexts, particularly considering the functional role of immune activation in elite HIV controllers.

Despite the observed inflammatory similarities, important biological differences between the two conditions must be acknowledged. In autoimmune diseases, inflammation primarily reflects dysregulated immune responses associated with loss of self-tolerance and progressive tissue injury [27,29]. In contrast, in elite HIV controllers, sustained innate immune activation may partially represent a functional antiviral state contributing to long-term viral suppression [7,17]. These findings may additionally reflect a condition of incomplete immune homeostasis, in which antiviral and inflammatory signalling pathways persist despite apparent clinical equilibrium.

Consequently, the concept of modulation appears more appropriate than broad immunological suppression. This distinction is particularly relevant because excessive attenuation of immune activation in elite HIV controllers could theoretically compromise mechanisms involved in viral control. Thus, the emerging hypothesis does not support identical therapeutic approaches across both conditions, but rather context-dependent strategies aimed at regulating inflammatory activity while preserving essential immunological functions. From this perspective, non-pharmacological interventions may represent a safer and more biologically adaptable approach for inflammatory modulation in both scenarios.

From an applied perspective, these findings allow the conceptualisation of a progressive framework of inflammatory modulation targeting the proposed shared inflammatory axis. At a first level, non-pharmacological interventions may function as baseline modulators of systemic inflammatory activity. At a second level, pharmacological strategies with indirect anti-inflammatory effects could be considered in selected contexts. Finally, targeted modulation of the interferon–inflammasome axis would represent a more intensive level of intervention, requiring careful evaluation of the risk–benefit balance, particularly in elite HIV controllers. Importantly, these considerations should be interpreted strictly within a hypothesis-generating framework and are not intended to guide clinical decision-making.

Validation of this hypothesis will require comparative studies integrating shared biomarkers such as IL-1β, IL-6, type I interferons, sCD14, and IP-10 across both conditions. In particular, longitudinal studies and controlled trials will be necessary to determine whether modulation of the proposed inflammasome–interferon axis may translate into clinical benefit without compromising essential immunological functions.

From a translational perspective, recognition of partially overlapping inflammatory mechanisms across distinct chronic immune-mediated conditions may contribute to improved biomarker stratification, earlier identification of inflammatory comorbidity risk, and the development of more individualised immunomodulatory strategies. Although these implications remain speculative, they reinforce the relevance of investigating shared innate inflammatory pathways across apparently distinct disease contexts.

Future research should prioritise longitudinal comparative studies integrating inflammatory biomarkers, transcriptomic signatures, and immunophenotypic profiling across both conditions. In particular, further investigation of inflammasome- and interferon-related pathways may help clarify whether these inflammatory patterns represent convergent adaptive responses, maladaptive chronic activation, or distinct biological processes with partial mechanistic overlap.

In summary, the chronic inflammatory activity observed in elite HIV controllers and autoimmune diseases does not appear to represent entirely isolated biological phenomena but rather partially overlapping inflammatory processes. On this basis, it is possible to propose the hypothesis that selective modulation of the inflammasome–interferon axis may represent a potential translational target. From a biological perspective, this convergence reflects activation of conserved innate immune pathways operating across distinct immune-mediated conditions, reinforcing the plausibility of shared inflammatory mechanisms.

Rather than constituting a definitive conclusion, this proposal opens a field of investigation that encourages reconsideration of inflammation as a biologically interconnected process across apparently distinct diseases. Nevertheless, the proposed convergence should be interpreted as a biologically plausible conceptual framework rather than definitive evidence of a unified inflammatory mechanism.

### Limitations

This study has several limitations that should be acknowledged. First, as a critical narrative and integrative reflection, the selection of evidence followed a purposive concept-driven approach, which may introduce selection bias and limit reproducibility despite efforts to maintain conceptual consistency.

Second, substantial heterogeneity exists across both elite HIV controllers and autoimmune diseases. Elite controllers represent a biologically diverse population, while autoimmune diseases encompass multiple conditions with distinct pathophysiological mechanisms. Accordingly, the proposed convergence should be interpreted as a conceptual framework rather than a uniform biological model.

Third, most available evidence derives from observational, translational, and mechanistic studies, with limited interventional data—particularly in elite HIV controllers. This restricts causal inference and limits the direct clinical applicability of the proposed hypotheses. Moreover, the biological role of immune activation differs between both conditions: whereas inflammation is predominantly pathogenic in autoimmune diseases, it may simultaneously contribute to viral control in elite HIV controllers, limiting direct therapeutic extrapolation.

Finally, direct comparative studies evaluating inflammatory mechanisms across both conditions within a unified analytical framework remain absent. Consequently, the proposed convergence is based on indirect evidence derived from parallel lines of research rather than head-to-head analyses. Nevertheless, this limitation also highlights the potential value of integrative conceptual approaches capable of identifying biologically plausible intersections across traditionally separate research fields.

## 5. Conclusions

This study provides an integrative perspective on the potential convergence between elite HIV controllers and autoimmune diseases, supporting the hypothesis of partially overlapping inflammatory mechanisms involving sustained innate immune activation and the inflammasome–interferon axis. Across both conditions, these pathways are associated with persistent production of pro-inflammatory mediators and chronic systemic inflammatory activity.

Beyond descriptive synthesis, this work proposes a conceptual framework in which chronic inflammation may be understood as a biologically interconnected process extending across apparently distinct immune-mediated conditions. Within this context, the inflammasome–interferon axis emerges as a plausible pathophysiological node susceptible to selective modulation.

Importantly, these findings do not support a unified therapeutic approach, but rather context-dependent modulation strategies. In elite HIV controllers, preservation of immune-mediated viral control remains essential, limiting direct extrapolation from autoimmune disease therapeutics. Nevertheless, both pharmacological and non-pharmacological interventions may represent areas for future investigation; however, their safety, efficacy, and potential impact on antiviral immune control require careful evaluation before any clinical application can be considered.

From a translational perspective, the proposed convergence supports further investigation into shared inflammatory biomarkers, longitudinal inflammatory trajectories, and targeted modulation strategies. However, these implications remain exploratory and require validation through comparative mechanistic studies and controlled clinical research.

Overall, this reflection supports a broader conceptualisation of chronic inflammation as a biologically interconnected phenomenon that may transcend traditional disease boundaries. By proposing a plausible convergence centred on innate immune activation and inflammasome–interferon signalling, this study opens new avenues for comparative and hypothesis-driven translational research across distinct immune-mediated conditions.

## Figures and Tables

**Table 1:** Comparative inflammatory features identified in elite HIV controllers and autoimmune diseases.

Domain	Elite HIV Controllers	Autoimmune Diseases	Shared Mechanistic Feature	Key References
Persistent innate immune activation	Sustained immune activation despite viral suppression and absence of ART	Chronic activation associated with loss of immune tolerance and dysregulated innate immune activation, particularly in interferon-driven systemic autoimmune diseases.	Persistent activation of innate immune pathways	[4,7,18]
Central interferon-driven inflammatory axis in lupus and related disorders	Increased interferon-stimulated gene expression and IFN-related biomarkers	Central interferon-driven inflammatory axis in lupus and related disorders	Sustained type I interferon activation	[25,26,27]
NLRP3 inflammasome activation	Inflammasome-related inflammatory signalling associated with chronic activation	NLRP3-mediated IL-1β and IL-18 production linked to tissue damage	Inflammasome-driven inflammation	[19,21,23]
Elevated inflammatory biomarkers	Increased sCD14, IP-10, IFN-γ, IL-6, D-dimer and sTNF-R1	Elevated TNF, IL-1, IL-6, IFN-γ and systemic inflammatory mediators	Persistent systemic inflammatory burden	[5,30,31,32]
Monocyte, macrophage, and NK-cell activation	Altered monocyte homeostasis and activated NK-cell phenotype	Activated innate immune cells amplify inflammatory cascades	Innate immune dysregulation	[9,10,12]
NF-κB and cytokine signalling	Persistent inflammatory signalling contributing to chronic activation	Amplification of inflammatory cascades and tissue injury	Cytokine-mediated inflammatory perpetuation	[24,28,29]
Chronic systemic inflammation	Residual inflammation associated with cardiovascular and metabolic risk	Persistent inflammation associated with inflammaging and multiorgan damage	Long-term inflammatory burden	[13,15,33]
Potential modulatory pathways	Exploratory evidence for anti-inflammatory modulation without compromising viral control	Targeted modulation of interferon and inflammasome pathways	Modulation of shared inflammatory pathways	[34,35,36,37,38]

## Data Availability

The data supporting the findings of this study are available from the corresponding author upon reasonable request.
